# Early Postoperative Weight Loss Predicts Weight Loss up to 5 Years After Roux-En-Y Gastric Bypass, Banded Roux-En-Y Gastric Bypass, and Sleeve Gastrectomy

**DOI:** 10.1007/s11695-022-06166-x

**Published:** 2022-07-16

**Authors:** Onno M. Tettero, Valerie M. Monpellier, Ignace M. C. Janssen, Ingrid H. M. Steenhuis, Maartje M. van Stralen

**Affiliations:** 1 Department of Health Sciences and Amsterdam Public Health Research Institute, Vrije Universiteit (VU University) Amsterdam, De Boelelaan 1105, 1081 HV Amsterdam, the Netherlands; 2 Nederlandse Obesitas Kliniek (Dutch Obesity Clinic), Huis ter Heide, Zeist, the Netherlands

**Keywords:** Bariatric surgery, Early weight loss, Long-term weight loss prediction, Intervention selection

## Abstract

**Purpose:**

Previous studies showed that patients with lower weight loss after bariatric surgery could be identified based on early postoperative weight loss. However, these studies had only 12–36-month follow-up. This study aimed to explore whether patients in the lowest weight loss quartile at 3 months had lower weight loss trajectories up to 5 years after Roux-en-Y gastric bypass (RYGB), banded Roux-en-Y gastric bypass (BRYGB), and sleeve gastrectomy (SG) surgery.

**Methods:**

Weight was assessed preoperatively, and 3, 6, 9, 12, 24, 36, 48, and 60 months postoperatively. Patients were grouped into four categories based on quartiles of percentage total weight loss (%TWL) at 3-month follow-up. Results were compared between the lowest %TWL quartile group and other quartile groups.

**Results:**

Patients underwent either RYGB (*n*=13,106; 72%), SG (*n*=3585; 20%), or BRYGB (*n*=1391, 8%) surgery. Weight loss trajectories of patients in the lowest %TWL quartile group remained lower than that of other quartile groups throughout a 5-year follow-up, for all three types of surgery. Patients in the lowest %TWL quartile group had higher age at surgery, higher baseline BMI, and were more likely to be male (in the SG group), and to suffer from diabetes, hypertension, dyslipidemia, and osteoarthritis.

**Conclusion:**

This study showed a positive association between weight loss at 3 and 12 to 60 months after bariatric surgery. Weight loss at 3 months after surgery could be used to identify patients whose anticipated weight loss trajectories are below average, to potentially improve their outcomes through early behavioral or medical interventions.

**Graphical Abstract:**

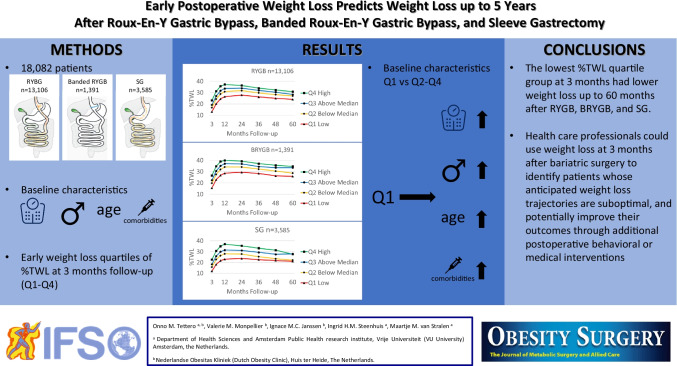

## Introduction

Bariatric surgery is the most effective treatment for patients with severe obesity, yet approximately 20% of patients experience weight loss lower than 50% of percentage excess weight loss (%EWL), or lower than 20–25% of percentage total weight loss (%TWL) 1–2 years post-surgery [1–3]. Lower weight loss may result in a continuance or recurrence of medical and psychological comorbidities, higher cancer incidence, cardiovascular events, cardiovascular deaths, and impaired health-related quality of life [4–9]. It is therefore essential to predict which groups of patients suffer from lower weight loss to increase weight loss through additional pre- or postoperative interventions.

Previous research showed that predictors of lower weight loss at 5 years after Roux-en-Y gastric bypass (RYGB) surgery include higher preoperative BMI kg/m^2^ and waist circumference, absence of laparoscopic surgery, higher age, unhealthy eating behavior, and psychological factors, such as low cognitive function, personality, and psychiatric disorders [10, 11]. Other previous research showed associations between early postoperative weight loss and nadir postoperative weight loss. However, these studies all had relative short- to medium-term weight loss outcomes (12 to 36 months) [12–18]. As obesity is a chronic disease, and as patients tend to regain weight from 2 years after surgery onwards, longer-term treatment outcomes of bariatric surgery are most meaningful [19, 20]. To our knowledge, only one small (*n*=130) retrospective study examined the association between early postoperative weight loss and long-term weight loss (i.e., 7 years after surgery), and found no relationship [21]. Furthermore, this study only examined weight loss after sleeve gastrectomy (SG) surgery. As weight loss variance differs between surgery types, weight loss after other types of surgery should also be examined [22].

The aim of this study was to explore whether patients in the lowest weight loss quartile at 3 months after RYGB, SG, or banded RYGB (BRYGB) surgery were more likely to have lower weight loss trajectories than other patients up to 5-year follow-up. In addition, differences in patient characteristics were compared between patients in the lowest weight loss quartile group at 3 months and other quartile groups.

## Materials and Methods

### Study Design and Setting

This multi-center prospective cohort study took place in the largest multicenter organization for treatment of obesity in the Netherlands, the Dutch Obesity Clinic (Nederlandse Obesitas Kliniek, NOK). Bariatric surgery (i.e., RYGB, BRYGB, or SG) takes place in one of the nine surgical centers throughout the Netherlands. The RYGB is the preferred surgical procedure in the obesity clinics, as this type of surgery shows higher long-term weight loss than the SG within all nine selected clinics. The RYGB is also preferred for patients who suffer from diabetes mellitus type 2. Furthermore, since 2012, one of the nine clinics performs the rather new BRYGB instead of the RYGB as even higher weight loss was expected after BRYGB surgery. Finally, the SG is preferred for patients with a preoperative BMI kg/m^2^ higher than 50. In most cases, a secondary procedure (single-anastomosis duodeno-ileal bypass (SADI)) is performed after SG to increase further weight loss.

Surgery is complemented with a comprehensive behavioral change program for over 5000 patients per year. Patients that qualify for treatment participate in six preoperative and 13 postoperative group sessions up to 1 year postoperatively. These group sessions are alternately supervised by a multidisciplinary team consisting of a psychologist, dietician, or physiotherapist. Along with these group sessions, patients also have five recurrent individual medical sessions with a bariatric physician. Patients who are unable to function in group sessions (e.g., due to insufficient Dutch language skills, or psychological issues) are offered individual treatment sessions instead. The care program aims to help patients adopt a new lifestyle, optimize weight loss in the first postoperative year, and maintain achieved weight loss in the long term. After the first postoperative year, patients have an annual consultation with a bariatric physician (and if necessary, with a psychologist and dietician) up to 5 years after surgery.

### Study Population

Follow-up data up to 5 years was collected prospectively up to April 2020 from patients who underwent primary RYGB, SG, or BRYGB surgery between January 2012 and April 2019. All patients combined their procedure with the group (91%) or individual (9%) pre- and postoperative care program. Patients who did not have a weight measurement 3 months postoperatively and at least one weight measurement 6, 9, 12, 24, 36, 48, or 60 months postoperatively were excluded from the study.

### Measures

Body weight was assessed preoperatively and 3, 6, 9, 12, 18, 24, 36, 48, and 60 months postoperatively. %TWL was calculated to express postoperative weight change between baseline and 3 (defined as early weight change), 6, 9, 12, 24, 36, 48, and 60 months of follow-up. The following formula was used to calculate %TWL:

$$\%\mathrm{TWL}=\frac{\mathrm{preoperative}\ \mathrm{weight}-\mathrm{current}\ \mathrm{weight}}{\mathrm{current}\ \mathrm{weight}}\times 100$$

Patients were grouped into four categories based on quartiles of %TWL at 3 months follow-up. The first quartile represents the 25% of patients with the lowest weight loss at 3 months. These %TWL quartile groups were named as follows:

- Low %TWL (i.e., first %TWL quartile patients)
- Below Median %TWL (i.e., second %TWL quartile patients)
- Above Median %TWL (i.e., third %TWL quartile patients)
- High %TWL (i.e., fourth %TWL quartile patients)

Sex, age, height, and comorbidities (i.e., type 2 diabetes, hypertension, dyslipidemia, osteo-arthritis, and obstructive sleep apnea syndrome) were registered at baseline. All measurements were assessed at the clinic by a physician (e.g., comorbidities) or another health care professional (e.g., weight, sex, age, and height).

### Statistical Analysis

All analyses were conducted for each type of surgery separately (i.e., RYGB, SG, and BRYGB). All continuous variables were visually inspected and tested for normality by the Shapiro-Wilk test. Patients’ characteristics that followed a normal distribution were defined by the mean and standard deviation. Nominal variables were defined by the number and percentage of cases. Descriptive statistics were used to summarize patients’ baseline characteristics: age, sex, BMI kg/m^2^, and comorbidities (i.e., type 2 diabetes, hypertension, dyslipidemia, osteo-arthritis, and obstructive sleep apnea syndrome). Baseline characteristics were described separately per surgery type and per %TWL quartile group. Baseline characteristics of the Below Median, Above Median, and High %TWL groups were compared to the Low %TWL group with *t* tests for continuous data and chi-square for nominal data. Patient retention rates were calculated to enable accurate interpretation of the TWL figures:

$$\%\mathrm{Patient}\ \mathrm{retention}\ \mathrm{rate}=\frac{\mathrm{number}\ \mathrm{of}\ \mathrm{weight}\ \mathrm{measurements}}{\mathrm{number}\ \mathrm{of}\ \mathrm{bariatric}\ \mathrm{procedures}}\times 100$$

To compare patients who did and who did not have a weight measurement at 5 years after surgery on baseline characteristics BMI kg/m^2^, sex, age, and comorbidities (diabetes, hypertension, dyslipidemia, osteoarthritis, and obstructive sleep apnea syndrome), *t* tests for continuous data and chi-square for nominal data were performed.

Weight change was assessed through a linear mixed model. In this model, we assessed how %TWL changed over time from 3 to 60 months after surgery. Results of patients in the Below Median, Above Median, and High %TWL group were compared to results of patients in the low %TWL group by adding these groups as an effect modifier. First, a crude model was developed in which a random slope and random intercept for patients were tested. Second, potential confounders were added to the model as fixed effects (i.e., age at surgery, sex, baseline BMI kg/m^2^, and number of comorbidities (1–5: type 2 diabetes, hypertension, dyslipidemia, osteo-arthritis, and obstructive sleep apnea syndrome)).

Analysis was performed using SPSS 25 (IBM Corp. Released 2017. IBM SPSS Statistics for Windows, Version 25.0. Armonk, NY: IBM Corp.), except for the linear mixed model, which was analyzed using STATA, version 13 (StataCorp. 2013. Stata 13 Base Reference Manual. College Station, TX: Stata Press). Findings were considered statistically significant if the *p* value was < 0.05.

## Results

### Study Population

A total of 19,422 patients were selected. Of these patients, 1340 (6.9%) were excluded from analysis as they did not have a weight measurement at 3 months and at least one weight measurement at 6, 9, 12, 24, 36, 48, or 60 months postoperatively. The study population consisted of 18,082 patients of which 13,106 (72%) underwent RYGB, 3585 (20%) underwent SG, and 1391 (8%) underwent BRYGB surgery. Characteristics of the study participants are described per %TWL quartile group, and per surgery type in Table 1.

**Table 1:** Characteristics of the study participants per %TWL quartile group at 3 months after surgery, presented as mean and standard deviation unless stated otherwise

	%TWL at 3 months after surgery^a^			
	Low	Below Median	Above Median	High
RYGB (***n***=13,106)	3277	3276	3277	3276
Age at surgery, years	47.2 ± 10.6	45.6 ± 10.6*	45.1 ± 10.4*	43.7 ± 10.5*
Female gender, % (*n*)	81.9% (2,684)	83.2% (2725)	82.1% (2689)	75.2 (2464)*
BL BMI kg/m^2^	44.1 ± 5.5	43.5 ± 5.1*	43.0 ± 4.9*	42.4 ± 4.5*
Diabetes, % (*n*)	29.0% (947)	23.5% (770)*	21.5% (705)*	20.7% (678)*
Hypertension, % (*n*)	43.8% (1428)	38.3% (1253)*	37.6% (1230)*	34.0% (1112)*
Dyslipidemia, % (*n*)	23.8% (778)	21.2% (695)*	20.0% (653)*	19.7% (646)*
OSAS, % (*n*)	14.4% (469)	15.1% (496)*	14.5% (476)	15.5% (508)
Osteoarthritis, % (*n*)	17.0% (555)	14.7% (482)*	13.0% (424)*	13.5% (443)*
SG (***n***=3585)	896	896	897	896
Age at surgery, years	43.5 ± 13.5	41.0 ± 12.9*	38.5 ± 12.6*	36.6 ± 11.8*
Female gender, % (*n*)	76.9% (689)	78.1% (700)	77.1% (692)	67.9% (608)*
BL BMI kg/m^2^	48.1 ± 7.7	47.2 ± 7.2*	46.2 ± 6.7*	45.2 ± 6.6*
Diabetes, % (*n*)	21.8% (195)	13.7% (123)*	12.6% (113)*	10% (89)*
Hypertension, % (*n*)	37.2% (332)	28.7% (257))*	27.9% (250)*	24.7% (221)*
Dyslipidemia, % (*n*)	18.1% (162)	13.9% (124)*	12.6% (113)*	9.8% (88)*
OSAS, % (*n*)	17.4% (155)	15.6% (140)	15.6% (140	15.2% (136)
Osteoarthritis, % (*n*)	12.5% (112)	10.2% (91)	7.8% (70)*	6.4% (57)*
BRYGB (***n***=1391)	347	348	348	348
Age at surgery, years	46.0 ± 11.5	44.2 ± 11.1*	43.0 ± 11.0*	42.0 ± 10.8*
Female gender, % (*n*)	78.1% (271)	76.4 (266)	67.7% (267)	71.8% (250)
BL BMI kg/m^2^	44.0 ± 6.6	43.2 ± 5.8	43.2 ± 5.5	42.3 ± 5.2*
Diabetes, % (*n*)	23.9% (79)	15.1% (50)*	18.2% (62)	17.0% (58)*
Hypertension, % (*n*)	40.0% (132)	28.6% (95)*	35.8% (122)	33.9% (116)
Dyslipidemia, % (*n*)	23.9% (79)	13.9% (46)*	19,4% (66)	17.3% (59)*
OSAS, % (*n*)	15.2% (50)	15.7% (52)	12,9% (44)	17.0% (58)
Osteoarthritis, % (*n*)	9.7% (32)	7.5% (25)	8,5% (29)	8.2% (28)

^a^Low, Below Median, Above Median, and High %TWL represents the 1st, 2nd, 3rd, and 4th quartile groups based on %TWL at 3 months after surgery

*BL* baseline, *BMI* body mass index, *OSAS* obstructive sleep apnea syndrome, *RYGB* Roux-en-Y gastric bypass, *SG* sleeve gastrectomy, *BRYGB* banded-RYGB

*Significant difference compared to the Low %TWL quartile group, *p* ≤ 0.05

First, there were differences in patient characteristics between the Low %TWL group and the other %TWL quartile groups. On average, patients in the Low %TWL group had a higher age at surgery, higher baseline BMI kg/m^2^, and were more likely to suffer from diabetes, hypertension, dyslipidemia, and osteoarthritis. Furthermore, there were fewer women in the Low %TWL group of patients that underwent SG surgery. Second, patient characteristics differed between surgery types: patients that underwent SG had higher age at surgery, were less likely to be female, had higher baseline BMI kg/m^2^, and were less likely to suffer from comorbidities (except for obstructive sleep apnea syndrome) than patients that underwent RYGB or BRYGB.

### Patient Retention Rates

Patient retention rates are described in Table 2. A comparison between patients who did or who did not have a weight measurement at 5 years after surgery showed that these groups have similar baseline characteristics, such as age, sex, BMI kg/m^2^, and several comorbidities (Table 3). Except that patients who did not have a weight measurement at 5 years after surgery were more likely to suffer from diabetes and hypertension at baseline.

**Table 2:** Patient retention rates per annual follow-up month and surgery type

Follow-up in months	12	24	36	48	60
Bariatric procedures					
Total (*n*)	18,082	14,070	10,460	6952	4289
RYGB *(n*)	13,106	10,524	8228	5721	3676
SG (*n*)	3585	2570	1581	827	406
BRYGB (*n*)	1391	976	651	404	207
TWL measurements					
Total % (*n*)	94% (16,945)	79% (11,039)	66% (6855)	53% (3691)	41% (1779)
RYGB % (*n*)	94% (12,350)	80% (8445)	67% (5517)	54% (3084)	42% (1543)
SG % (*n*)	92% (3296)	72% (1843)	60% (941)	50% (827)	37% (151)
BRYGB % (*n*)	93% (1299)	77% (751)	61% (397)	49% (404)	41% (85)

*TWL* total weight loss, *RYGB* Roux-en-Y gastric bypass, *SG* sleeve gastrectomy, *BRYGB* banded-RYGB

**Table 3:** Characteristics of the study participants who did or did not have a weight measurement at 5 years after surgery, presented as mean and standard deviation unless stated otherwise

	Patients with a weight measurement at 60 m	Patients without a weight measurement at 60 m
RYGB, % (n)	42% (1543)	58% (2133)
Age at surgery, years	45.7 ± 10.2	45.2 ± 10.8*
Female gender, % (*n*)	81.9% (1884)	80.0% (9457)*
BL BMI kg/m^2^	43.8 ± 5.3	43.2 ± 5.0*
Diabetes, % (*n*)	26.2% (603)	23.4% (2744)*
Hypertension, % (*n*)	43.1% (990)	37.7% (4425)*
Dyslipidemia, % (*n*)	22.5% (518)	21.2% (2486)
OSAS, % (*n*)	13.7% (314)	15.1% (1779)
Osteoarthritis, % (*n*)	15.2% (350)	14.6% (1716)
SG﻿, % (n)	37% (151)	63% (255)
Age at surgery, years	45.8 ± 10.2	45.2 ± 10.8*
Female gender, % (*n*)	78.1% (196)	74.6% (2650)
BL BMI kg/m^2^	47.5 ± 8.6	46.6 ± 7.1
Diabetes, % (*n*)	19.9% (201)	14.3% (3035)*
Hypertension, % (*n*)	43.0% (143)	28.6% (2529)*
Dyslipidemia, % (*n*)	17.9% (206)	13.6% (3060)
OSAS, % (*n*)	13.1% (218)	15.8% (2983)
Osteoarthritis, % (*n*)	15.9% (211)	8.7% (3233)*
BRYGB﻿, % (n)	41% (85)	59% (122)
Age at surgery, years	44.9 ± 10.7	43.5 ± 11.3
Female gender, % (*n*)	76.6% (131)	75.3% (995)
BL BMI kg/m^2^	43.5 ± 5.6	43.3 ± 5.9
Diabetes, % (*n*)	24.6% (107)	17.8% (1041)*
Hypertension, % (*n*)	45.1% (78)	33.2% (846)*
Dyslipidemia, % (*n*)	27.5% (103)	17.6% (1044)
OSAS, % (*n*)	14.1% (20)	14.9% (189)
Osteoarthritis, % (*n*)	11.3% (16)	8.0% (101)*

*BL* baseline, *BMI* body mass index, *OSAS* obstructive sleep apnea syndrome, *RYGB* Roux-en-Y gastric bypass, *SG* sleeve gastrectomy, *BRYGB* banded-RYGB

*Significant difference compared to patients with a weight measurement at 60 m, *p* ≤ 0.05

### Weight Change Per %TWL Quartile Group

**Fig. 1:**
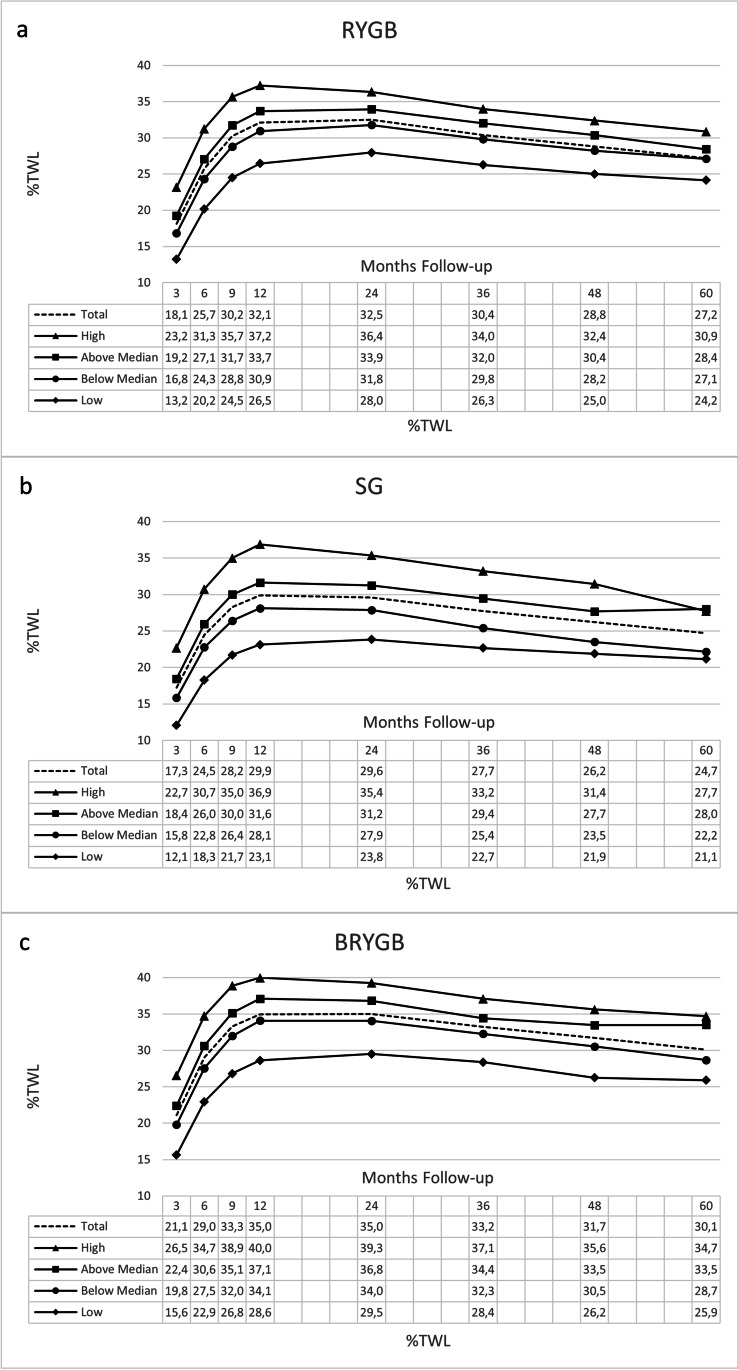
**a**–**c** Weight change trajectories per percentage total weight loss quartile group at 3 months postoperatively (low, below median, above median, and high) for primary Roux-en-Y gastric bypass (RYGB), sleeve gastrectomy (SG), and banded Roux-en-Y gastric bypass (BRYGB) presented as mean percentage total weight loss (%TWL)

Weight change trajectories of the %TWL quartile groups are shown in Figure 1a–c. While the patients’ weight change trajectory remained the lowest in the Low %TWL group after 3 months postoperatively, weight change trajectories were higher in the other three %TWL quartile groups. This was similar for RYGB, SG, and BRYGB. Graphs with individual weight loss lines for patients in the Low %TWL group are shown separately for RYGB, SG, and BRYGB in Appendix Fig. 2.

Table 4 shows the results of the linear mixed models of weight change, comparing the Below Median, Above Median, and High %TWL groups to the Low %TWL quartile group for RYGB, SG, and BRYGB. These models were adjusted for age, sex, baseline BMI kg/m^2^, and the number of baseline comorbidities (1–5: type 2 diabetes, hypertension, dyslipidemia, osteoarthritis, and obstructive sleep apnea syndrome). In all three models, the adjustment of confounders did not lead to relevant changes compared to the crude model (the crude model is shown in Appendix Table 5). The unstandardized regression coefficient (B) represents the change in %TWL between two time-points (i.e., 3 months versus 6, 9, 12, 24, 36, 48, or 60 months) while comparing the Low %TWL quartile group to the other %TWL quartile groups (i.e., Low versus Below Median, Low versus Above Median, and Low versus High %TWL quartile groups). For example, in the RYGB group, the regression coefficient for 60MQ2 is −1.03%TWL (Table 4). This means that the difference in %TWL between the Low and the Below Median groups from 3 to 60 months is −1.03%TWL. This is also visible in Figure 1a, which shows that the change in %TWL from 3 to 60 months is 11% in the Low group and 10% in the Below Median group (i.e., a difference of approximately one percentage point). Thus, the Low %TWL group shows lower weight loss throughout 5-year follow-up, but less weight regains (i.e., negative regression coefficients in Table 4) in the mid-to long-term follow-up compared to the other groups, especially after BRYGB surgery.

**Table 4:** Linear mixed model results of weight change in patients who underwent RYGB, SG, or BRYGB surgery ^a^

%TWL	RYGB					SG					BRYGB				
	*B*	95% CI			*p* value	*B*	95% CI			*p* value	*B*	95% CI			*p* value
6MQ2	0.64	0.37	-	0.90	<0.001	0.79	0.26	-	1.33	<0.001	0.41	−0.15	-	1.47	0.108
6MQ3	0.96	0.69	-	1.22	<0.001	1.39	0.86	-	1.93	<0.001	0.41	0.25	-	1.87	0.011
6MQ4	1.19	0.92	-	1.45	<0.001	1.91	1.38	-	2.45	<0.001	0.41	0.25	-	1.88	0.010
9MQ2	0.86	0.59	-	1.13	<0.001	1.04	0.50	-	1.59	<0.001	0.42	0.33	-	1.98	0.006
9MQ3	1.36	1.09	-	1.63	<0.001	2.06	1.51	-	2.60	<0.001	0.42	0.87	-	2.53	<0.001
9MQ4	1.39	1.12	-	1.66	<0.001	2.83	2.28	-	3.38	<0.001	0.42	0.62	-	2.26	0.001
12MQ2	0.93	0.66	-	1.20	<0.001	1.30	0.76	-	1.84	<0.001	0.41	0.56	-	2.18	0.001
12MQ3	1.27	1.01	-	1.54	<0.001	2.30	1.76	-	2.84	<0.001	0.41	0.96	-	2.58	<0.001
12MQ4	0.93	0.66	-	1.19	<0.001	3.18	2.64	-	3.72	<0.001	0.41	−0.25	-	1.37	0.176
24MQ2	0.35	0.06	-	0.63	0.019	0.52	−0.09	-	1.13	0.093	0.45	−0.34	-	1.44	0.226
24MQ3	0.11	−0.18	-	0.40	0.444	1.33	0.72	-	1.94	<0.001	0.45	−0.26	-	1.52	0.166
24MQ4	−1.33	−1.62	-	−1.04	<0.001	1.39	0.78	-	1.99	<0.001	0.45	−1.94	-	−0.16	0.021
36MQ2	−0.07	−0.40	-	0.25	0.664	-0.47	−1.24	-	0.29	0.224	0.57	−1.81	-	0.42	0.224
36MQ3	−0.20	−0.53	-	0.13	0.230	0.79	0.03	-	1.56	0.041	0.58	−1.90	-	0.36	0.180
36MQ4	−2.11	−2.44	-	−1.78	<0.001	0.32	−0.43	-	1.08	0.400	0.57	−3.57	-	−1.34	<0.001
48MQ2	−0.45	−0.84	-	−0.07	0.021	−1.49	−2.52	-	−0.46	0.005	0.77	−2.09	-	0.91	0.442
48MQ3	−0.70	−1.09	-	−0.31	<0.001	0.05	−0.98	-	1.09	0.920	0.73	−1.64	-	1.24	0.787
48MQ4	−2.49	−2.88	-	−2.09	<0.001	−0.64	−1.66	-	0.38	0.217	0.74	−3.99	-	−1.09	0.001
60MQ2	−1.03	−1.51	-	−0.54	<0.001	−1.54	−3.10	-	0.02	0.052	0.92	−3.16	-	0.44	0.138
60MQ3	−1.49	−2.00	-	−0.99	<0.001	0.59	−1.01	-	2.19	0.470	0.95	−1.51	-	2.20	0.718
60MQ4	−3.10	−3.62	-	−2.57	<0.001	−3.06	−4.65	-	−0.15	<0.001	1.01	−3.32	-	0.64	0.184
Constant	7.90	6.96	-	8.83	<0.001	11.14	9.85	-	12.42	<0.001	10.29	8.10	-	12.48	<0.001

^a^Analyses were adjusted for age, sex, baseline BMI KG/M2, and number of baseline comorbidities (1–5: type 2 diabetes, hypertension, dyslipidemia, osteoarthritis, and obstructive sleep apnea syndrome)

*RYGB* Roux-en-Y gastric bypass, *SG* sleeve gastrectomy, *BRYGB* banded Roux-en-Y gastric bypass (BRYGB), *%TWL* percentage total weight loss, *M* months after surgery, *Q* quartile based on %TWL at 3 months after surgery, *B* unstandardized regression coefficient, *CI* confidence interval

## Discussion and Conclusion

This study demonstrated that patients in the lowest TWL-quartile at 3 months after RYGB, SG, and BRYGB surgery (i.e., Low %TWL quartile group) had lower weight loss trajectories up to 5 years after surgery than other patients, similar for all three types of surgery. In addition, we found that patients in the lowest TWL-quartile at 3 months after surgery had significantly higher baseline age and BMI kg/m^2^ and were more likely to be male (in the SG group), and to suffer from diabetes, hypertension, dyslipidemia, or osteoarthritis, than other patients.

Even though we recognize that long-term weight loss prediction is multifactorial, our results suggest that weight loss at 3 months may play a significant role in predicting weight loss 5 years after surgery. Short-term weight loss might be an indication of the “effectiveness” of bariatric surgery for a specific patient. Although patients in the Lowest %TWL quartile group were more likely to have lower weight loss up to 5 years, it seems that this group also had a more stable weight over time (i.e., less weight regain) than patients in the Below Median, Above Median, and High %TWL quartile groups. Our finding that patients in the lowest TWL-quartile at 3 months is associated with lower weight loss trajectories in up to 5 years after surgery is in line with previous studies that found positive associations between short- and medium-term postoperative weight loss (i.e., 12–36 months after surgery) [12–18]. For example, Mor et al. found that patients in the lowest %EWL quartile at 1 month were more likely to remain in the lowest quartile at 12 months, while patients in the lowest quartile at 12 months were more likely to remain in the lowest %EWL quartile at 36 months [14]. Others found strong positive associations between weight loss up to 6 months and short-term outcomes [12] and maximum weight loss [13].

The added value of this study beyond these previous studies is the follow-up up to 60 months after surgery, which is much longer than the follow-up of 12, 24, and 36 months of previous studies. These longer-term treatment outcomes of bariatric surgery are most meaningful as obesity is a chronic disease. Longer-term treatment outcomes are also important since patients tend to regain weight after approximately 2-year follow-up [19, 20]. For example, Barhouch et al. found that weight regain affects only 5.7% of patients 2 years after surgery, but up to 75.6% at 7 years after surgery [10]. Studies with shorter follow-up periods are therefore limited to account for the impact of subsequent weight regain on comorbidities, mortality, and health-related quality of life [19]. In contrast to the current study, the only other study we found that examined the association between short- and long-term weight loss after bariatric surgery did not find a significant association [21]. In this other study, 130 patients were assessed up to 7 years after surgery. Four weeks after surgery, the predictor used in this other study, may have been too early to predict long-term weight loss. Ideally, a time-point for intervention selection should be long enough after surgery to be predictive, yet soon enough after surgery to intervene at the earliest possible time in the patients’ weight loss trajectory. In addition, intervention selection should not happen to soon after surgery, when weight loss is still mainly determined by the surgery and barely by other factors that could be improved through intervention. Further research is needed to determine the most optimal postoperative time-point to predict long-term weight loss.

Our finding that patients in the Low %TWL quartile group had a higher age and BMI kg/m^2^ at baseline, and were more likely to be male (in the SG group), and to suffer from comorbidities, confirms previous research [23]. This group may have received bariatric surgery too late. If these patients had received bariatric surgery when they were eligible, they might have been less prone to preoperative weight gain and obesity-related comorbidities. Further research is needed to explore why some patients do not receive bariatric surgery when they are eligible. Previous research suggests reasons that could play a role. For example, despite the effectiveness of bariatric surgery, the majority of eligible patients are not interested in surgery due to the perceived risk [24]. Furthermore, physicians’ concerns about complications following bariatric surgery may result in low referral rates to bariatric surgery [25]. In some countries, waiting lists could also be a factor (e.g., USA 159 days, Spain 397 days, and Canada up to 5 years, versus 30 days for the population of this study) [26–28]. Finally, some people with severe obesity may not be aware of the option of bariatric surgery. Further research is also needed to examine how to reach people that qualify for bariatric surgery, so they can consider treatment at an earlier stage, preventing unnecessary weight gain and obesity-related comorbidities.

Based on our finding that the lowest TWL-quartile at 3 months is associated with lower weight loss trajectories up to 5 years after surgery, patients in the lowest TWL-quartile at 3 months after surgery could be selected for additional postoperative interventions to optimize their weight loss. This patient group may particularly benefit from improvement in weight loss, as patients in the Low %TWL quartile group had a higher baseline BMI kg/m^2^ and were more likely to suffer from diabetes, hypertension, dyslipidemia, and osteoarthritis. A great benefit of short-term weight loss as predictor is that weight assessments are quick and easily accessible. Further research is needed into why some patients have lower early weight loss to determine which interventions most effectively increase weight loss and reduce comorbidities. Behavioral lifestyle interventions in the first months after bariatric surgery focusing on physical activity and eating behavior have shown to improve weight loss after bariatric surgery [29]. Furthermore, recent studies suggest that biological interventions may enhance weight loss in some patients. For example, gut hormones are regulators of energy homeostasis and drivers for eating behavior and have shown to be important mediators for weight loss after RYGB and SG surgery. Pharmacotherapeutic strategies that target these gut hormones are therefore a new promising approach to addressing low early weight loss after bariatric surgery [30].

Positive aspects of this study are the multicenter aspect and substantial sample size, resulting in large statistical power. Furthermore, all measurements (e.g., weight, comorbidities, and other data) were assessed at the clinics by health care professionals and were therefore more reliable than self-reported measurements by people that suffer from obesity [31]. Finally, this study examined associations for different surgery types, which enables readers to distinguish predictive models for RYGB, SG, and BRYGB. A limitation of this study is that patient retention rates decrease with each subsequent follow-up year. It may leave patients that are lost to follow-up underexposed. Long-term follow-up in bariatric surgery is generally low in studies with large populations. However, a comparison within this study population between patients who did and who did not have a weight measurement at 5 years after surgery showed that these groups have similar baseline characteristics. Therefore, interpretation of %TWL figures is increasingly limited with each subsequent follow-up year. Furthermore, other outcomes than weight loss, such as predicted reduction of comorbidities, may also have additional value for intervention selection. The current study used %TWL quartiles to distinguish different groups of patients. Results show that even patients in the Low %TWL quartile group lost weight at 5-year follow-up (RYGB 24.2%TWL, SG 21.1%TWL, and BRYGB 25.9%TWL), which may still be sufficient to reduce metabolic diseases [32]. Further research is needed to determine optimal early postoperative %TWL levels that can be used to select patients for interventions aiming to reduce metabolic diseases through weight loss.

## Conclusion

This study showed a positive association between short-term weight loss and weight loss up to 5 years after RYGB, SG, and BRYGB surgery. Data about short-term weight loss is easily accessible and can be used to identify patients whose anticipated weight loss trajectories are below average. The outcomes of these patients might then be improved through additional postoperative behavioral or medical interventions, thereby preventing continuance or recurrence of obesity-related comorbidities, mortality, and impaired health-related quality of life.

## Figures and Tables

**Fig. 2:**
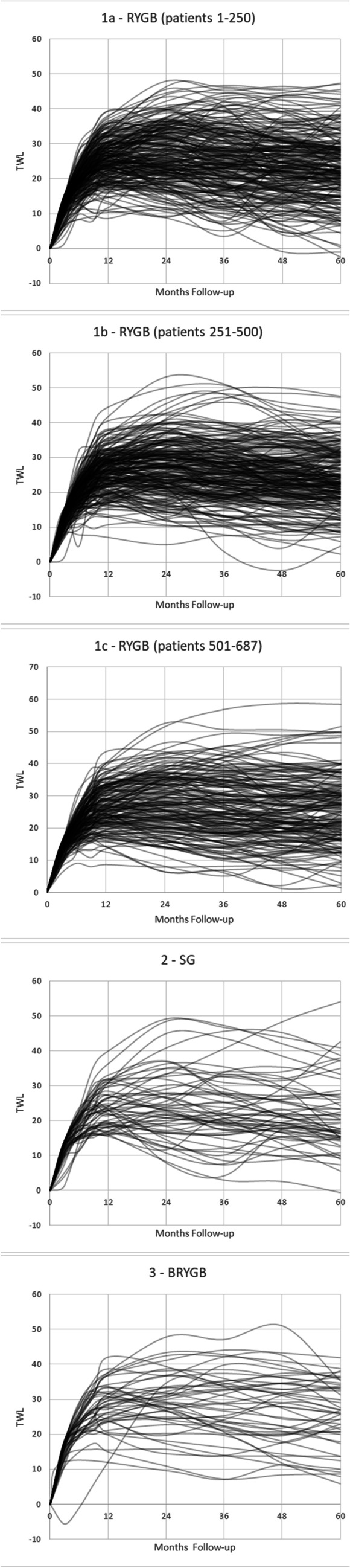
Individual weight loss lines for patients in the Low %TWL group for RYGB, SG, and BRYGB

## Appendix

### Appendix B

**Table 5:** Crude model: Linear mixed model results of weight change in patients who underwent RYGB, SG, or BRYGB surgery ^a^

TWL	RYGB					SG					BRYGB				
	*B*	95% CI			*p* value	*B*	95% CI			*p* value	*B*	95% CI			*p* value
6m Q2	0.63	0.36	-	0.89	<0.000	0.77	0.23	-	1.30	0.005	0.66	−0.15	-	1.47	0.108
6m Q3	0.94	0.68	-	1.21	<0.000	1.37	0.84	-	1.91	<0.000	1.06	0.25	-	1.87	0.011
6m Q4	1.18	0.91	-	1.44	<0.000	1.89	1.35	-	2.42	<0.000	1.07	0.25	-	1.88	0.010
9m Q2	0.85	0.58	-	1.12	<0.000	1.00	0.45	-	1.55	<0.000	1.16	0.33	-	1.98	0.006
9m Q3	1.35	1.08	-	1.62	<0.000	2.02	1.47	-	2.57	<0.000	1.70	0.87	-	2.53	<0.000
9m Q4	1.37	1.10	-	1.64	<0.000	2.79	2.24	-	3.34	<0.000	1.44	0.62	-	2.26	0.001
12m Q2	0.91	0.65	-	1.18	<0.000	1.25	0.71	-	1.79	<0.000	1.37	0.56	-	2.18	0.001
12m Q3	1.25	0.99	-	1.52	<0.000	2.26	1.72	-	2.80	<0.000	1.77	0.96	-	2.58	<0.000
12m Q4	0.91	0.64	-	1.17	<0.000	3.14	2.59	-	3.68	<0.000	0.56	−0.25	-	1.37	0.176
24m Q2	0.34	0.05	-	0.63	0.020	0.44	−0.17	-	1.05	0.154	0.55	−0.34	-	1.44	0.226
24m Q3	0.10	−0.19	-	0.39	0.497	1.26	0.65	-	1.87	<0.000	0.63	−0.26	-	1.52	0.166
24m Q4	−1.33	−1.62	-	−1.04	<0.000	1.32	0.71	-	1.93	<0.000	−1.05	−1.94	-	−0.16	0.021
36m Q2	−0.08	−0.41	-	0.25	0.633	−0.57	−1.34	-	0.19	0.141	−0.69	−1.81	-	0.42	0.224
36m Q3	−0.22	−0.54	-	0.11	0.194	0.72	−0.04	-	1.48	0.065	−0.77	−1.90	-	0.36	0.180
36m Q4	−2.12	−2.45	-	−1.79	<0.000	0.25	−0.51	-	1.01	0.518	−2.45	−3.57	-	−1.34	<0.000
48m Q2	−0.46	−0.85	-	−0.07	0.019	−1.64	−2.67	-	−0.61	0.002	−0.59	−2.09	-	0.91	0.442
48m Q3	−0.72	−1.12	-	−0.33	<0.000	−0.07	−1.11	-	0.96	0.891	−0.20	−1.64	-	1.24	0.787
48m Q4	−2.51	−2.91	-	−2.11	<0.000	−0.76	−1.78	-	0.26	0.142	−2.54	−3.99	-	−1.09	0.001
60m Q2	−1.02	−1.51	-	−0.54	<0.000	−1.61	−3.17	-	−0.05	0.043	−1.36	−3.16	-	0.44	0.138
60m Q3	−1.52	−2.03	-	−1.02	<0.000	0.55	−1.05	-	2.16	0.499	0.34	−1.51	-	2.20	0.718
60m Q4	−3.12	−3.65	-	−2.59	<0.000	−3.06	−4.65	-	−1.48	<0.000	−1.34	−3.32	-	0.64	0.184
Constant	13.17	12.6	-	13.7	<0.000	12.12	11.43	-	12.82	<0.000	15.6	1.50	-	1.62	<0.000

^a^Analyses were not adjusted for potential confounders

*RYGB* Roux-en-Y gastric bypass, *SG* sleeve gastrectomy, *BRYGB* banded Roux-en-Y gastric bypass, *%TWL* percentage total weight loss, *M* months after surgery, *Q* quartile based on %TWL at 3 months after surgery, *B* unstandardized regression coefficient, *CI* confidence interval
